# Intersectionality and underrepresentation among health care workforce: the case of Arab physicians in Israel

**DOI:** 10.1186/s13584-015-0004-0

**Published:** 2015-04-15

**Authors:** Yael Keshet, Ariela Popper-Giveon, Ido Liberman

**Affiliations:** Western Galilee Academic College, POB 2125, Akko, 24121 Israel; David Yellin Academic College, 3a Avraham Granot St., Jerusalem, 93706 Israel; Western Galilee Academic College, POB 2125, Akko, 24121 Israel

**Keywords:** Arabs, Intersectionality, Israel, Medicine, Underrepresentation

## Abstract

**Background:**

An intersectionality approach that addresses the non-additive influences of social categories and power structures, such as gender and ethnicity, is used as a research paradigm to further understanding the complexity of health inequities. While most researchers adopt an intersectionality approach to study patients’ health status, in this article we exemplify its usefulness and importance for studying underrepresentation in the health care workforce. Our research objectives were to examine gender patterns of underrepresentation in the medical profession among the Arab minority in Israel.

**Methods:**

We used both quantitative and qualitative methodologies. The quantitative data were obtained from the *2011 Labor Force Survey* conducted by the Israeli Central Bureau of Statistics, which encompassed some 24,000 households. The qualitative data were obtained through ten semi-structured, in-depth interviews conducted during 2013 with Arab physicians and with six nurses working in Israeli hospitals.

**Results:**

The findings indicate that with respect to physicians, the Arab minority in Israel is underrepresented in the medical field, and that this is due to Arab women’s underrepresentation. Arab women’s employment and educational patterns impact their underrepresentation in medicine. Women are expected to enter traditional gender roles and conform to patriarchal and collectivist values, which makes it difficult for them to study medicine.

**Conclusions:**

Using an intersectionality approach to study underrepresentation in medicine provides a foundation for action aimed at improving public health and reducing health disparities.

## Background

### Intersectionality theory

Intersectionality theory originated in the work of African American feminist scholars (such as [1-3]). These scholars studied multiple forms of marginalization, which are mutually constituted and cannot be understood by approaches that treat race/ethnicity and sex/gender as distinct subjects of inquiry. “The intersectional experience is greater than the sum of racism and sexism, any analysis that does not take intersectionality into account cannot sufficiently address the particular manner in which Black women are subordinated” ([2]: 140).

The intersectional approach differs both from unitary analyses and from multiple approach analyses. Unitary analyses focus on gender, ethnicity or socioeconomic status separately. Multiple approach analyses focus on more than a single category but operate according to an additive assumption that treats multiple marginalization as distinct categories that can be layered [4]. Intersectionality is not an additive approach; it does not calculate the cumulative impact of social positions and structural forces such as gender, race and class, as the sum of their independent effects. Alternatively, it focuses on examining how social positions and forces interact, beyond their additive impacts, to shape and influence human experiences [5].

The intersectional approach, which addresses the non-additive influences of gender and ethnicity, facilitates the study of health and disease at different intersections of identity, social position, processes of oppression or privilege, policies and institutional practices [6]. In the literature of public health, intersectionality is identified as an important theoretical framework [7], and is used as a research paradigm to further understanding of the complexity of health inequities [5,6,8]. Intersectionality has much to offer to the research of public health by providing more precise identification of inequalities, developing intervention strategies and ensuring that findings are relevant within specific populations [6].

Use of the intersectionality approach in health research has focused largely on patients’ illnesses and treatments, as well as on inequalities in health [9]. Studies focus, for example, on cancer screening in Canada [10], immunization in India [11], and experiences of disability and HIV in Zambia [12]. In this article, however, we suggest that it may be used to study underrepresentation and the intersection of ethnicity and gender in the health care workforce, and specifically among physicians. Intersectionality among physicians has implications for health inequalities. Identifying intersectional patterns of underrepresentation in the medical profession indicates “points for health intervention” ([13]: 150), which provide a foundation for action aimed at improving public health and reducing health disparities by means of ensuring ethnic and gender diversity in the health system.

### Ethnic diversity within the health care workforce

Ethnic diversity within the health care workforce is considered to play an important role in reducing health disparities among different ethnic populations. One of the factors that influence health inequities is underrepresentation in health care professions. The term ‘underrepresented in medicine’ refers to those racial and ethnic minority populations that are underrepresented in the medical professions relative to their numbers in the general population [14]. Since ethnic minority populations are underrepresented among health professionals [15,16], broadening the ethnic diversity of the health care workforce can contribute to achieving high-quality health care that is accessible, equitable and culturally competent [17-19].

Enhancing health professionals’ diversity can afford minority patients from populations underrepresented in the health professions greater opportunity to consult practitioners of their own ethnic background, since minority health professionals disproportionately serve minority and other medically underserved groups, thereby improving access to care for vulnerable populations [20,21]. Increasing diversity among health professionals also means that there are more professionals who are familiar with the language and culture of patients, thereby improving communication, comfort level, trust and partnership among patients and practitioners, which leads to better use of appropriate health care and adherence to effective programs, resulting in improved health outcomes. Patient-practitioner language concordance is associated with better interpersonal care, greater medical comprehension and greater likelihood of keeping follow-up appointments, particularly important in primary care and mental health settings. Minority physicians also introduce into the formal health care system different norms and habits associated with their own culture, thereby creating an atmosphere of ethnic diversity and mutual familiarity among health care staff members.

Thus, greater health care workforce diversity may lead to improved access to care for underserved populations and better interpersonal interactions between patients and health professionals and thus improved public health [18]. Consequently, minority recruitment to the health professions is used as a strategy to address ethnic disparities in health care [19,22]. However, improved health care is a function not only of language and culture concordance, but also of patient-practitioner gender relations.

### Gender characteristics within the health care workforce

Ethnic diversity within the health care workforce is not the only factor that impacts quality of care. Gender dyads–gender correspondence between patient and physician – may also enhance the provision of patient-centered care as well as patient-physician communication, which has been associated with improved patient’s health [23].

A systematic review of literature concerning the impact of gender dyads on clinician–patient communication [24] identified variances between the ways that gender is enacted within the four gender dyad combinations. Differences are evident in the patients’ agendas elicited, what is talked about, communication style, exhibition of power and status and the length of consultation. Male physician/male patient dyads and female physician/female patient dyads are characterized by relative ease and equality between physicians and patients, compared with opposite sex dyads [25]. In female physician/female patient dyads there is more psychosocial talk and also more bio-medical talk than in any other dyad combination. These dyads are characterized by the most encouraging communication style; both verbal (through positive statements and encouraging back channel responses) and nonverbal (nodding). Female physician/female patient dyads are also characterized by a calmer tone of voice, suggesting relative ease in interactions, and produce the longest consultations in which more talk occurs than in any other dyad combination [24]. Gender concordance in female physician/female patient dyads demonstrates significantly more patient-centered care [23]. Female patients seen by female physicians have been observed to have the highest patient-centered scores for their visits, compared to female patients who are treated by male physicians and male patients who consult with either female or male physicians [26].

Gender concordance in female physician/female patient dyads is especially important in gynecology. A review of recent studies of women seeking gynecological or obstetrical care points to the importance of the physician’s gender in relation to the patient’s preferences, differences in communication style and patient’s satisfaction [27]. Most female patients preferred a female rather than a male gynecologist–obstetrician. This was partly explained by the more patient-centered communication style employed by female gynecologists–obstetricians.

More generally, research shows that patients prefer to be seen by physicians of similar ethnic and gender backgrounds; thus, a diverse physician pool serves patient interests [28,29]. Since both ethnic and gender characteristics of the health care workforce seem to influence the quality of health care, especially among minority populations, we turned to study this intersectionality among the Arab minority in Israel.

### The Arab minority in Israel

The Arab minority is the largest ethnic minority in Israel, comprising about 20% of the population. Arabs and Jews in Israel differ in religion, culture and language. The Arab minority’s way of life is still semi-traditional, and it is far less modern and secular than the dominant Jewish culture, despite a degree of modernization [30]. The Arab population’s socioeconomic status is low relative to the Jewish majority population [31]. Unemployment rates among Arabs in Israel are higher, education levels are lower and income is lower. Their mean subjective measure of social status is also far lower compared to that of the Jews [32]. Although they enjoy full citizenship, Arabs in Israel are largely an underprivileged minority with a history of disadvantage in income, education and employment [33]. In addition, the ongoing national conflict in the region and the inferior political status of the Arabs in Israel place tremendous pressures on this population [34].

Israel’s National Health Law, enacted in 1995, is based on equality in the provision and quality of care for all citizens, including the Arab citizens. Nevertheless, health disparities between Arabs and Jews persist. Mortality and morbidity among the Arab population are higher than among the Jewish population and life expectancy is lower [35]. It appears that many of these differences may be attributed to Arabs’ overall lower socioeconomic status and to some extent also to subjective measures of social status. While both objective socioeconomic status and subjective measures of social status seem to explain disparities in physical health between Arab and Jewish men, they do not explain the disparities in physical health between Arab and Jewish women [32].

In addition to objective socioeconomic status and subjective measures of social status, cultural factors such as values, norms and traditions can explain health disparities of ethnic minorities, such as the Arabs in Israel. In recent years, the Arab population in Israel has experienced a rapid and marked process of westernization in lifestyle, which has led to a significant increase in the incidence of both diabetes and cancer [36]. The mortality rate associated with diabetes among the Arab population is twice as high as that among the Jewish population. This can be explained mainly by cultural factors such as unhealthy diet and lack of physical activity [36].

Gender variables likewise influence the health of the Arab minority in Israel. Arab women in Israel are a minority within a minority. They endure dual marginalization—being women within the Arab patriarchal society and belonging to an ethnic minority in the dominantly Jewish nation state of Israel [31,37]. Certain chronic diseases are more prevalent among Arab than Jewish women. For example, they display far higher rates of diabetes, lower survival rates from cancer [36], and they are less likely to practice healthy behavior or comply with medical recommendations [35]. The transition from traditional to Western style eating habits is manifested in a higher incidence of obesity among the Arab population, particularly among older women. About 70% of Arab women aged 55–64 suffer from obesity compared with 36.4% among Jewish women of the same age [36].

Daoud [38] identified six major obstacles to good health among Arab women: an unhealthy lifestyle, compliance with patriarchal norms, a rapid transition in lifestyle, the political situation, low socioeconomic status and limited access to specific health care services. Cultural restrictions, and specifically the subordination of women in Arab-Islamic society and men’s control of women’s behavior, were cited as obstacles to good health. The prohibition of jogging in public, for example, illustrates how patriarchal attitudes perpetuate the lower status of women. Women cannot jog in public because they are forbidden to attract the attention of strangers or be “visible” in public [39]. Arab women’s acquiescence to men and significant others in their community constitutes a significant part of “the Arab woman’s identity,” according to which women are primarily committed to their family’s welfare rather than to their own [40].

These studies’ findings highlight the need to develop culturally competent and more accessible health care services for Arab women in Israel. In the present article, we focus on gender representation of Arab female physicians in the Israeli public health system, which is one of the factors that could contribute to establishing competent and accessible health care services for Arab women in Israel. While this study addresses specifically Arab women in Israel, it has more general implications for women in other ethnic minority groups as well.

Our research objectives were to examine gender patterns of underrepresentation in medicine and to point to some of the main reasons for this underrepresentation in health care services, such as gendered education and employment patterns among the Arab minority in Israel. By doing so, we seek to exemplify the usefulness and the importance of the intersectionality approach (e.g., the intersection of ethnicity and gender) to the study of underrepresentation in medicine. In the long run, a better understanding of the intersection of gender and ethnicity in the health care professions may contribute to the enhancement of accessible health services for Arab women in Israel.

## Methods

We used both quantitative and qualitative methodologies to obtain two diverse but related and complementary types of data. To examine the intersection of gender and ethnicity in the health care services in Israel, we used quantitative methodology. The quantitative data were obtained from the Labor Force Survey. This is a major survey conducted by the Israeli Central Bureau of Statistics among a large sample of households that are part of Israel’s permanent population. The survey monitors the development of the labor force in Israel, its size and characteristics, as well as the rate of unemployment and other trends. We used data from the 2011 Labor Force Survey, which encompassed about 24,000 different households [41]. In each household, one questionnaire relating to information pertaining to the entire household, and to each member of family aged 15 and above, was completed. The survey covered a total of 101,838 people. We used data regarding employees in the health care services sector. Statistical analysis was performed using the Chi-square test and the z-test for comparison of proportions.

In order to learn about Arab physicians’ perspectives concerning gender and ethnicity in medicine, we used a qualitative method. The qualitative data were obtained in ten semi-structured, in-depth interviews with Arab physicians working in Israeli hospitals conducted during 2013. We focused on hospitals located in two large Israeli cities – Haifa and Jerusalem – that serve mixed Jewish and Arab populations. Despite the fact that some 50% of Arab physicians are community doctors, as compared with 38% of Jewish physicians [42], we chose to focus on Arab physicians who are employed in public hospitals in mixed cities, where they treat both Arab and Jewish patients. We assumed that this unique setting could better contribute to the understanding of the Arab physicians’ issues of integration and alienation in a multicultural context. Moreover, these physicians are striving to attain senior professional positions and in these positions the gender aspect is more prominent. We interviewed nine men and one woman, a ratio reflecting the small number of female physicians in the Arab population in Israel. Seven were specialists in anesthesia, internal medicine, pediatrics (2) and trauma (2), while the remaining three were currently at various stages of specialization – one in pediatrics and two in internal medicine.

The interviews focused on the unique experiences of the Arab physicians as an ethnic minority group within the Jewish dominated Israeli health system. We asked the physicians about their decision to study medicine, about their experiences with patients and colleagues, both Jews and Arabs; about the gender aspects of their work and the special needs of their Arab patients.

We furthermore sought to understand why Arab women seldom take up medicine even though many of them choose to work in the health field, mainly in nursing. We therefore interviewed six nurses as well: four female nurses and two male nurses employed in Israeli public hospitals, who serve a mixed (Jewish and Arab) population. We asked the nurses about their decision to study nursing rather than medicine, about the importance of Arab female physicians, about the needs of Arab patients, and why they thought that there are far less Arab women physicians than men. All the interviews were recorded, transcribed verbatim and analyzed thematically by the first two authors.

Thematic analysis [43,44] was used to identify key themes. Thematic analysis is widely used to identify, analyze and report patterns or themes within qualitative data, in order to detect repeated patterns of action and meaning. This analysis was conducted by reading through the transcripts to develop a set of codes according to which the structure of the data was organized. Codes were applied to common words and phrases in order to condense the data. The codes were then arranged according to higher level categories and analyzed to identify relationships between them. The analysis involved moving back and forth between the entire data set and the coded extracts of the data.

## Results

### Quantitative findings: Arab women are underrepresented in medicine while Arab men are not

The data from the Labor Force Survey [41] indicate that the Arab population in Israel is underrepresented in medicine. Physicians comprise only 0.34% of the Arab population (aged 15 and above), in comparison to 0.51% of the Jewish population (also aged 15 and above) (P < .001). This pattern of underrepresentation, however, does not characterize the entire Arab population, but specifically Arab women. Jewish male physicians comprise 0.54% of the Jewish male population; Jewish female physicians comprise 0.49% of the Jewish female population, and Arab male physicians comprise a similar percentage (0.52%) of the Arab male population. Yet Arab female physicians comprise only 0.14% of the Arab female population (P < .001) (Table 1).

**Table 1:** **Underrepresentation in medicine: physicians in relation to others among the entire population by ethnicity and gender**

	**Physicians**		**Others**		**Comparison**	
	**(n = 504)**		**(n = 101550)**			
	**No.**	**%**	**No.**	**%**	**d.f**	***χ*** **2**
Jews	426	0.51%	82328	99.49%	1	10.63 *
Arabs	64	0.34%	19020	99.66%		
Men	278	0.54%	50966	99.46%	1	7.09 *
Women	226	0.43%	52584	99.57%		
Jewish men	219	0.54%	40220	99.46%	3	25.90 **
Jewish women	207	0.49%	42108	99.51%		
Arab men	51	0.52%	9767	99.48%		
Arab women	13	0.14%	9253	99.86%		

* *p* < .01. ** *p* < .001.

**Figure 1:**
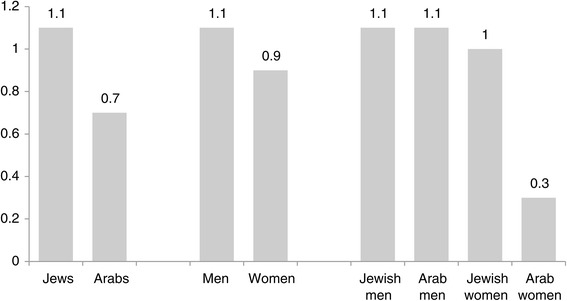
**Underrepresentation among physicians: population groups that are underrepresented relative to their numbers in the general population (N = 103,550)**.

Another way to reflect underrepresentation in medicine is by calculating the rate of Arab physicians in the entire population of Israeli physicians, in relation to the rate of Arabs in the general population. According to this calculation method, a complete representation is 1. We found that the rate of Arab physicians in the entire population of physicians, relative to their rate in the general Israeli population is 0.7, compared with 1.1 among the Jews. This general underrepresentation of Arabs in medicine is due to the underrepresentation of Arab women in medicine. The rate of Arab women physicians in the entire population of physicians, compared with the rate of Arab women in the general Israeli population is 0.3, compared to 1.1 among Jewish and Arab men, and 1.0 among the Jewish female population (Figure 1).

Arab women are therefore underrepresented in medicine, as compared to Arab men. This underrepresentation occurs despite the fact that the health care services sector provides a common employment path for women in Israel, and especially for Arab women. The percentage of Arab women employed in the health care services sector is higher than the percentage of Jewish women employed therein and that of Jewish and Arab men (10.1% vs. 8.4%, 2.5% and 3.2% respectively; P < .001) (Table 2). Why, then, do we find a lack of Arab female physicians?

**Table 2:** **Employees in the health care sector compared to other employees among all employees, by ethnicity and gender**

	**Employed in the health care sector**		**Otherwise employed**		**Comparison**	
	**(n = 2713)**		**(n = 47386)**			
	**No.**	**%**	**No.**	**%**	**d.f**	***χ*** **2**
Jews	2310	5.5%	39953	94.5%	1	3.51
Arabs	316	4.9%	6133	95.1%		
Men	689	2.6%	25654	97.4%	1	850.21 *
Women	2024	8.5%	21732	91.5%		
Jewish men	516	2.5%	20333	97.5%	3	840.20 *
Jewish women	1794	8.4%	19620	91.6%		
Arab men	152	3.2%	4673	96.8%		
Arab women	164	10.1%	1460	89.9%		

* *p* < .001.

The underrepresentation of Arab women in medicine is due to several factors, the chief of which are unique patterns of employment and education. Employment rates among Arab women in Israel are lower than those among Israel’s other population groups. While 51.6% of Jewish men, 50.6% of Jewish women, and 49.1% of Arab men are employed, only 17.5% of Arab women are employed (P < .001) (Table 3).

**Table 3:** **Employed people compared to those not employed among the entire population, by ethnicity and gender**

	**Employed (48,712)**		**Unemployed (53,126)**		**Comparison**	
	**No.**	**%**	**No.**	**%**	**d.f**	***χ*** **2**
Jewish men	20849	51.6%	19590	48.4%	3	3772.58*
Jewish women	21414	50.6%	20901	49.4%		
Arab men	4825	49.1%	4993	50.9%		
Arab women	1624	17.5%	7642	82.5%		

* *p* < .001.

A second factor is associated with educational patterns. The rate of academics among Arab women is lower than the rates of academics among Jews (both women and men) but is higher than the rate of academics among Arab men (11.69% vs. 27.22%, 22.56% and 9.79% respectively; P < .001). However, a major factor within the sphere of Arab education is the low rate of Arab women who hold a third degree (PhD or MD). While 6.2% of academic Jewish men, 2.7% of academic Jewish women, and 5.6% of academic Arab men have a third degree, only 0.7% of academic Arab women have a third degree (P < .001) (Tables 4 and 5).

**Table 4:** **Academics versus non-academic population, by ethnicity and gender**

	**Non-Academics (71,182)**		**Academics (20,775)**		**Comparison**	
	**No.**	**%**	**No.**	**%**	**d.f**	***χ*** **2**
Jewish men	29098	77.44%	8478	22.56%	3	1719.57*
Jewish women	28581	72.78%	10687	27.22%		
Arab men	7454	90.21%	809	9.79%		
Arab women	6049	88.31%	801	11.69%		

* *p* < .001.

**Table 5:** **Holders of first and second degrees compared to third degree graduates among all academics, by ethnicity and gender**

	**1** ^**st**^ **and 2** ^**nd**^ **(19,905)**		**3** ^**rd**^ **(870)**		**Comparison**	
	**No.**	**%**	**No.**	**%**	**d.f**	***χ*** **2**
Jewish men	7951	93.8%	527	6.2%	3	170.78*
Jewish women	10395	97.3%	292	2.7%		
Arab men	764	94.4%	45	5.6%		
Arab women	795	99.3%	6	.7%		

* *p* < .001.

Quantitative analysis of the ethnic aspect points, therefore, to the underrepresentation of Arab physicians in the Israeli health care system. From the ethnic point of view, both Arab men and Arab women should have been underrepresented equally. Nevertheless, intersecting the ethnic aspect with the gender aspect – following the intersectionality approach – reveals unique findings: only Arab women are underrepresented. While Arab women indeed participate in the health care workforce, and the health care services sector is a common employment path for them, their employment rate and the rate of PhDs among them are relatively very low. By contrast, Arab male physicians are well represented in the health care system.

### Qualitative findings: medicine as a profession for outstanding Arab men and flag bearing Arab women

Arab men’s prominent role as physicians in the Israeli health care system is intriguing. Since minority groups, in general, tend to be underrepresented in the medical professions [15,16], we sought to figure out the attraction of medicine to Arab men.

We interviewed Arab physicians working in Israeli hospitals located in two large Israeli cities – Haifa and Jerusalem – which have mixed Jewish and Arab populations. We began the interviews with the Arab physicians by asking: “What did you dream of being when you grow up?” Only three of the ten participants reported that they dreamed of being physicians: “I dreamed of being a doctor, especially in a kind of emergency room, performing operations.” The remainder, surprisingly, paints entirely different pictures. Muhammad wanted to be a journalist, Omar a literature teacher and Elias an architect, recalling that he loved to draw. Ahmed told us, “at first I had no idea and later when computers arrived, IT information technology, I thought about it.“ Although the Arab doctors we interviewed did not claim to have dreamed of a medical career, they did indicate that their parents had hoped they would become physicians: “I think it’s the dream of all Arab families, even if not everyone succeeds at it. Although in recent times there are people who think otherwise, everyone dreams that his son will become a doctor” (Muhammad).

Health professions constitute a sought-after and popular education and employment pathway among the Arab minority in Israel. More than half of Arab parents who desire a specific profession for their children want their children to become doctors, compared to roughly a quarter of parents in the majority Jewish population (Popper-Giveon A, Keshet Y: “It’s every family’s dream”: Choice of a medical career among the Arab minority in Israel. Submitted). Many Arab families in Israel, mostly from the middle and upper classes, encourage and even press their children to practice medicine. This profession is perceived as guaranteeing a good (high and steady) income and especially considerable respect among Jews and Arabs alike.

Israeli Arabs are perceived as a minority whose ethnicity and nationality are associated with populations and states that are in a state of conflict with Israel. Thus, while the literature points to the barriers minorities face when trying to integrate into scientific studies and professions such as medicine [45], those Arabs who choose or are pressed by their families to practice medicine do so specifically to address the unique challenges they confront in the Israeli labor market, which places restrictions on the Arab minority for security reasons.

The Arab physicians’ choice to follow medicine as a career is presented as an informed and practical choice. It constitutes a path to channel excellence among young Arabs whose exam scores are particularly high. Young Arabs are often sent to study abroad (among Israeli-born Jewish physicians, 85% studied medicine in Israel, compared to only 23% among the Arab physicians) [42,46]. Study abroad allows individual Arabs to distance themselves from the conflictual situation as an Arab minority in the dominantly Jewish State of Israel.

Choosing medicine appears furthermore to offer a path to social and economic mobility for the Arab minority in Israel. Because of the shortage of physicians in the country, this profession ensures a steady income. Medicine likewise conveys a respectable social status, especially in the Arab society. Moreover, the practice of medicine is described as being rooted in the desire to be integrated into dominant Jewish Israeli society. The process of integration begins already during studies and continues at the workplace, particularly in public hospitals that provide services to both Arab and Jewish patients.

Medicine, therefore, is a popular education and employment pathway among the Arab minority in Israel. However, as it emerged from the interviews, there is a difference between the aspirations of Arab men and those of Arab women. Talented Arab male academics are urged by their families to study and practice medicine. Exceptional Arab academic women, on the other hand, are channeled to teaching in their local communities (see also: (Popper-Giveon A, Keshet Y: “It’s every family’s dream”: Choice of a medical career among the Arab minority in Israel. Submitted), [47]).

In most Arab communities, where the way of life is still semi-traditional, far less modern and secular than the dominant Jewish culture [30], many Arab women face barriers when they apply to higher education [47,48]. Women are expected to fulfill traditional gender roles and obey patriarchal and collectivist values, and are thus channeled toward feminine professions, such as education and nursing. In the interviews with the nurses, the issue of traditional gender roles came to the fore. When we asked Zainab why talented Arab women seldom took up medicine, she replied:

I think because of the expectations that women should have a family life. I wanted to study medicine, but everyone asked me, “are you prepared to give up your life; not have a family life?” At some point it affected me, and I came round to their way of thinking. And now I look at women physicians, Arab and Jewish, who work at the hospital, and it’s hard, it’s very hard. I do not regret I chose to be a nurse and I am not going to continue on to medical studies.

Other nurses also emphasized the obligations of Arab women to conform to what are regarded as “feminine priorities”:

To imagine my life revolving only around academic studies is difficult. It seems to me that women think twice [about studies]… They want to get married, want to have children, a family, so it is better for us not to study. Starting a family, getting married, making money, things like that are important (Ahlam).

Medicine is hard work. Some doctors I see here (in the hospital) every day, from morning to night. I wonder if they spend some time with their family, if they sleep at all, if they rest. So if this is too much for a man, what about women? In our society (in Arab society), the woman needs to take care of the children, to be at home, with the family (Zariffa).

A woman’s decision to study medicine is also impacted by the attitudes of men in the society. They require a cooperative and supportive husband. Jamil, a male nurse, explains the traditional point of view:

A woman chooses to marry someone who will be responsible for her, while the man takes the initiative, is the active one. Medicine requires seven years of study, then specialization, and so on and so forth. During all this time the woman has to be the initiator. She does not choose to have a family but rather chooses to move forward and learn. This is rare.

A further factor that explains the small number of Arab women physicians is the attitude of Arab parents toward their daughters’ higher education. Although three of the female nurses interviewed mentioned that their parents did encourage them to study medicine, this is not always the case. Jamil, a male nurse, explained: “According to tradition if a woman is a virgin and not married, her parents are afraid (that she will besmirch the family’s honor). So they try to marry her when she is still young, and this prevents her from choosing to study medicine.” Jamil emphasized that “nursing jobs are seen as women’s work and the work of doctors is seen as manly … it seems to me this is because of tradition.”

Hence medicine, in particular, is not perceived as a suitable profession for Arab women. It requires long years of study and training, which overlap with the period during which women are expected to marry, bear children and devote themselves to their family. Moreover, Arab medical students generally study abroad, far from the supervision of the family.

Arabs find it difficult to meet the conditions of admission to medical school in Israel. So they choose to study abroad, and more men than women choose to study abroad. Let’s say, 90% men and 10% women are studying medicine abroad (Jamil, a male nurse).

It is easier for the Arab family to send her son to study medicine abroad than to send their daughter (Samir, a male nurse).

The outcome is that Arab and Jewish male physicians are similarly represented in the Israeli health system, but the representation of Arab women physicians remains very low. Only few Arab women physicians are viewed as flag-bearers, paving the way through the public health system in Israel.

Underrepresentation of Israeli Arab women in medicine mainly impacts Arab women patients. When we asked the Arab men physicians who work in public hospitals in Israel about the importance of Arab women physicians, they all referred to the importance of Arab female gynecologists but did not expand on this issue. In an interview with an Arab woman physician named Basma, however, the issue of Arab women physicians came to the fore.

It always bothered me that Arab women are mistreated… They are not so educated, not academics. They do not receive proper treatment. They are diagnosed with cancer only after a few years because they do not complain. I think that in other population groups it wouldn’t have happened… A lot of Arab women do not receive the right treatment. They do not speak Hebrew and the woman physician is Jewish and does not speak Arabic, so they go to her but they do not understand properly and do not follow her instructions. The physicians do not understand their complaints and they do not understand how to follow the treatment properly. They are not treated well.

Basma, as well as the nurses we interviewed, explains that Arab women experience communication difficulties with both Arab and Jewish male physicians. Arab women, especially the observant ones, feel more comfortable with Jewish women physicians (especially regarding women’s health issues) but here the language barrier comes into play. Basma claims that in general, Arab women connect with women who speak their language and who understand them more easily than with men. Basma’s and the nurses’ remarks indicate the importance for Arab women in Israel to be treated by an Arab female physician. This is crucial in gynecology, but also affects the health care experience in other cases, such as oncology and diabetes. Some men also prefer their wife to be treated by an Arab female physician, as Jamil, a male nurse, explained:

I personally, when I take my wife to the doctor, I would rather take her to a female doctor, not a male doctor. Religious women often choose female doctors, even for general checkups. If we do not find a female doctor then we have no option but to go to a male doctor. But the priority is a female doctor. For example if, God forbid, my wife would come to the emergency room, I would rather that a female doctor or nurse take care of her, not a male nurse or doctor. This is the first priority. But if it’s an urgent life threatening case, I would have no choice and a male doctor would treat her.

Nonetheless, despite the importance of Arab women physicians to Arab women’s health, the quantitative findings indicate, as aforementioned, that Arab women in Israel are underrepresented in medicine. As an educational and occupational route of mobility, medicine is reserved for outstanding Arab men, and only the flag-bearers among Arab women enter this profession, a fact that impacts the overall health condition of Arab women in Israel.

Basma, the Arab woman physician, presents herself in the interview as a pioneer who is paving the way, as someone who makes the effort to carve out a path for other Arab women to continue her footsteps:

I help Arab women, that’s my goal. That is what I really want and I hope I do. Not just in the health system, but in society as a whole. I wish to show young women that there is more than just getting married and having children. Many women tell me ’we see you and learn from you and then we also want to study.’

As a representative of the first generation of Arab women physicians in Israel, Basma’s remarks are important since they shed light on the particular realities of Arab women in Israel – physicians and patients alike.

## Discussion

The Arab minority in Israel is underrepresented in medicine. Physicians comprise only 0.34% of the Arab population in comparison to 0.51% of the Jewish population (p < .001). We can compare this underrepresentation to the situation among nurses (an issue that warrants further research), for example. In nursing there is no underrepresentation (0.62% vs. 0.69%, ns) (all data for a population aged 15 and above).

Yet this study reveals that Arab’s underrepresentation in medicine, with respect to physicians, is generated more by gender than by ethnically related issues. The findings indicate that the Arab’s underrepresentation is due to Arab women’s underrepresentation. This is contrasted to Arab male physicians, who are well represented in medicine as physicians. The difference we found is significant: Jewish male and female physicians, as well as Arab male physicians, comprise 0.54%, 0.49% and 0.52% of the relevant population, but Arab female physicians comprise only 0.14% of the Arab female population. By comparison - in nursing, the absence of underrepresentation can also be explained by gender factors. Nursing is perceived as a feminine occupation and in Israel far more women (1.2% of the population of women) than men (0.2% of all men) are nurses. The rate of Arab female nurses (0.8% of the population of Arab women) is lower than that of Jewish female nurses (1.2%), but the gap is far smaller than among female physicians. Moreover, we find a higher rate of Arab (0.4%) than Jewish (0.1%) men who are nurses (P < .001).

Why are the rates of Arab women physicians so low? Employment and educational patterns among Arab women impact their underrepresentation in medicine. General employment rates among Arab women in Israel are still relatively low. Moreover, while the health care services sector (such as nursing) is a common employment path for Arab women, the low rates of Arab women who hold a third degree constitute another major factor in their underrepresentation in medicine.

Arab women in Israel suffer dual marginality as women in patriarchal Arab society and as a minority in Israeli society. These impediments restrict their opportunities to find employment. Arab women tend to participate less in the Israeli work force, compared to other groups in the population. In general, they are still expected to fulfill first and foremost the roles of wife and mother [49]. Tasks that are performed indoors are generally perceived to be appropriate for women [50]. Although women are increasingly expected to acquire education and to work outside the home, their income is still considered to be complementary to that of the man, and the woman continues to be responsible for the care of the children and home [51]. More than men, they are expected to follow traditional norms and to conform to cultural values that constrain women.

Specifically, Arab women rarely tend to choose (and are seldom encouraged by their families) to study medicine and work as physicians. It seems that the long years of education and training required to make a career in medicine deter Arab women from choosing this path. Arab women, especially those from traditional families, are not permitted to stay overnight away from their home or village. In Israel, where many Arab students find it hard to integrate within prestigious universities and are forced to study medicine abroad (in Italy, Slovakia or neighboring Arab countries) [42,46], it becomes even more difficult for Arab women to study medicine.

The underrepresentation of Arab women in medicine can therefore be seen as the result of wider processes of selective modernization that Arab society in Israel has recently been undergoing [(Keshet Y, Popper-Giveon A, Liberman I: Selective modernization leads to intersectionality: Health professions as a pathway to employment for Arabs in Israel. Submitted)]. Arab society in Israel is undergoing deep processes of Westernization and modernization. These foreign values are adopted primarily by men (and mainly in the mixed cities, such as Jerusalem or Haifa). Women, on the other hand, are encouraged to follow tradition. Even women who enter higher education are frequently forced to return to their villages after graduation [47]. Most of these Arab women academics become teachers, generally in Arab schools. Teaching is perceived as an appropriate profession for women and a very high rate of academic Arab women are employed in education (Keshet Y, Popper-Giveon A, Liberman I: Selective modernization leads to intersectionality: Health professions as a pathway to employment for Arabs in Israel. Submitted): this occupation can be easily combined with housekeeping and child rearing, and involves little contact with Arab and especially Jewish men. Teaching in the Arab education system likewise offers women a steady job within the local community, far from Jewish population centers.

Along with teaching, the health care sector is also a common employment path for Arab women, and employs a relatively greater proportion of Arab women compared to other occupations. Yonay and Kraus [52] found that eight to ten percent of Arab Christian and Muslim women with post-secondary education in the labor force are nurses, and they constitute a majority of Arab women in professional jobs. However, as our findings indicate, as educational requirements increase, the representation of Arab women in the higher levels of the health care sector declines. The profession of physician, as demonstrated, constitutes a prevalent employment path for academically inclined Arab men but not for their female counterparts.

In-depth interviews conducted with Arab physicians demonstrate that the study of medicine presents a path to channel excellence that is available to the Arab minority in Israel; as well as a way to help reduce health disparities between the Jewish majority and the Arab minority populations (Popper-Giveon A, Keshet Y: “It’s every family’s dream”: Choice of a medical career among the Arab minority in Israel. Submitted). The altruistic ambition to “help Arab women” articulated by Basma is not reflected in the motives of Arab men physicians, which were found to be more practical. Their decision to study medicine appears to be impacted by family pressures, as well as by the desire for socioeconomic mobility and for integration in modern Israeli society. These motives apply primarily to Arab men and are less relevant to Arab women, who are usually expected to follow accepted gender roles and to comply with traditional norms and values [51].

## Conclusions

Arab men are thus adequately represented among Israeli physicians, whereas Arab women suffer from underrepresentation in medicine. Consequently, despite the recognized importance of culturally and gender competent health care, an Arab woman is highly unlikely to be treated by an Arab woman doctor. The present situation limits the possibility of forming Arab female patient/female physician dyads, thereby reducing the extent of culturally competent patient-centered care, which may significantly impact the quality of care available to Arab patients and its outcomes.

The present article also contributes to the use of the intersectionality approach in health disparities research. It demonstrates that intersectionality theory is a useful approach for studying underrepresentation in medicine and diversity in the health care workforce. By using the intersectionality approach, which transcends race/ethnicity as static social positions, the article draws attention to differences within and similarities across the various ethnic minority and majority populations.

Intersectionality has been recognized as a valuable research paradigm for furthering understanding of the complexity of health inequities [5,8,53-58]. Intersectionality challenges approaches that privilege any specific axis of inequality, such as race, class or gender, and emphasizes the potential of varied and fluid configurations of social locations and interacting social processes in the production of inequities. While the fields of gender and health have begun to engage with the theoretical and methodological insights of intersectionality, the alignment of intersectionality with the issue of underrepresentation in medicine remains largely uninvestigated.

In the present article, we stress the importance of examining such underrepresentation in the health care workforce using the theoretical approach of intersectionality. Bird et al. [13] have criticized the intersectionality approach, claiming that it fails to provide a sufficient foundation for action aimed at improving public health and reducing health disparities, and that it is “not intended to identify points for health intervention” ([13]: 150). Drawing on the data regarding the representation of physicians from the Arab minority in Israel, we propose that the intersectional approach be used as a foundation for action aimed at reducing health disparities through enhancing ethnic-gender diversity in health care professions, thereby improving the population’s health.

### Policy implications

Much research has been conducted on ways to encourage minorities to enter a medical career [59,60]. Gabard [60] argued that educational programs should be more aggressive in recruiting minority students through affirmative action, outreach to school-age children, community involvement and advertisements. Early exposure activities through partnerships between school systems, higher education institutes, community organizations, social leadership and private enterprises can be utilized to create “pipelines” for minority entry into health professional schools. In the case of Arab women in Israel, such activities should be oriented toward female Arab pupils at high schools. Issues of education, socialization and practical solutions to women’s roles in the family should receive special attention. The role of the fathers, specifically, who were found to be key agents of encouragement for Arab female students [48], should be recognized. Designated activities should be conducted among them, expanding their awareness and involvement in their daughters’ academic education.

In order to reduce the underrepresentation in medicine of the Arab population in Israel and to encourage culturally and gendered competent healthcare, special effort should be devoted to encourage talented Arab women to study medicine, and specifically gynecology. Consideration should be given to providing special educational routes for Arab women, which could lead to an increase in the number of Arab women physicians who would serve, among others, the population of Arab women patients.

### Research limitations and future research

This research has several limitations. One is the static nature of this study. The quantitative data represent only one year. Future research could address trends in other years, both backward and forward, since recent years have seen important changes in the Israeli Arab community with regard to the education and employment of women. Another limitation is the small number of interviewed women physicians. Future research should interview Arab women, both physicians and patients, in order to give them a voice and to flesh out the experience of gender as embedded in the cultural context as well as in power relations.

Yet another limitation is that we examined the physicians en bloc and did not differentiate between medical specialties. Future research could extend the use of intersectionality approach and underrepresentation among physicians to examine stratification in the medical profession. A deeper examination of the intersection of gender and ethnicity is needed to clarify the patterns of medical specialization generally, and the rates of Arab women in gynecology specifically. Previous research has shown, for example, that women are disproportionately represented within less prestigious specialties, such as pediatrics, psychiatry, obstetrics and gynecology [61-63]. Studying the intersection of gender and ethnicity may reveal a more complex picture. A study of U.S. medical students explored how factors intrinsic and external to medical education influence specialization patterns among black and white medical school graduates. The data suggest that a degree of racial division in medical specialization endures, but that this division does not neatly map onto specialty prestige and is deeply gendered. Black graduates were found to be more likely to enter high-prestige surgical residency programs than their white colleagues, but this finding pertains only to male medical school graduates [64]. Further research is therefore needed to study the effect of intersectionality of ethnicity and gender on medical specialization and stratification. Understanding the intersectional patterns of medical specialization and stratification may help to encourage male and female medical students from minority groups to enter prestigious medical fields such as surgery, thereby improving public health in multi-cultural contexts.
